# Comparison of different classification systems for pulmonary nodules: a multicenter retrospective study in China

**DOI:** 10.1186/s40644-023-00634-y

**Published:** 2024-01-22

**Authors:** Feipeng Song, Qian Yang, Tong Gong, Kai Sun, Wenjia Zhang, Mengxi Liu, Fajin Lv

**Affiliations:** 1 Department of Radiology, The First Affiliated Hospital of Chongqing Medical University, No.1 YouYi Road, Chongqing, 400010 China; 2 Department of Radiology, Hubei Cancer Hospital, Wuhan, China; 3 Department of Radiology, Sichuan Provincial People’s Hospital, Chengdu, China; 4 Department of Radiology, The Second Hospital of Shanxi Medical University, Taiyuan, China

**Keywords:** Tomography, X-ray computed, Pulmonary nodule, Category

## Abstract

**Background:**

To compare the diagnostic performance of Lung-RADS (lung imaging-reporting and data system) 2022 and PNI-GARS (pulmonary node imaging-grading and reporting system).

**Methods:**

Pulmonary nodules (PNs) were selected at four centers, namely, CQ Center (January 1, 2018-December 31, 2021), HB Center (January 1, 2021–June 30, 2022), SC Center (September 1, 2021–December 31, 2021), and SX Center (January 1, 2021–December 31, 2021). PNs were divided into solid nodules (SNs), partial solid nodules (PSNs) and ground-glass nodules (GGNs), and they were then classified by the Lung-RADS and PNI-GARS. The sensitivity, specificity and agreement rate were compared between the two systems by the χ^2^ test.

**Results:**

For SN and PSN, the sensitivity of PNI-GARS and Lung-RADS was close (SN 99.8% vs. 99.4%, P < 0.001; PSN 99.9% vs. 98.4%, P = 0.015), but the specificity (SN 51.2% > 35.1%, PSN 13.3% > 5.7%, all P < 0.001) and agreement rate (SN 81.1% > 74.5%, P < 0.001, PSN 94.6% > 92.7%, all P < 0.05) of PNI-GARS were superior to those of Lung-RADS. For GGN, the sensitivity (96.5%) and agreement rate (88.6%) of PNI-GARS were better than those of Lung-RADS (0, 18.5%, P < 0.001). For the whole sample, the sensitivity (98.5%) and agreement rate (87.0%) of PNI-GARS were better than Lung-RADS (57.5%, 56.5%, all P < 0.001), whereas the specificity was slightly lower (49.8% < 53.4%, P = 0.003).

**Conclusion:**

PNI-GARS was superior to Lung-RADS in diagnostic performance, especially for GGN.

**Supplementary Information:**

The online version contains supplementary material available at 10.1186/s40644-023-00634-y.

## Background

Cancer is the leading cause of death in China and developed countries [1, 2] and the second leading cause of death in the USA [3]. Lung cancer is projected to become the leading cause of cancer death in China and the USA by 2022 [4]. Therefore, the prevention, early detection and early treatment of lung cancer are particularly important. Chest CT plays an irreplaceable role in the early detection and diagnosis of lung cancer. Many studies [5–7] show that chest CT in lung cancer screening can significantly reduce the mortality of lung cancer.

How to manage and evaluate the malignancy risk of pulmonary nodules (PNs) detected by lung cancer screening is a primary issue for radiologists and clinicians. Accordingly, the Lung imaging-reporting and data system (Lung-RADS) version 1.0 was released in 2014 by the American College of Radiology (ACR) and was updated in 2022 [8]. As a framework for chest CT screening reports for the evaluation and management of PNs, Lung-RADS performed well in clinical practice [9, 10]. However, with the widespread use of this system, many studies revealed its low sensitivity and indicated that the Lung-RADS could underestimate the risk of malignancy of such nodules and had poor predictive ability, especially for nonsolid nodules [11, 12]. In addition, the imaging signs of nodules were ignored by Lung-RADS when focusing on the classification by diameter or volume, and the description of additional imaging features in category 4X was not specific, resulting in many inconsistencies in the determination of the 4X category and a certain false-positive rate in clinical application [13, 14].

Lung-RADS was based on data from people in Western countries, but the incidence of lung cancer and pulmonary infectious diseases among Asian people, including Chinese people, differs due to the influence of different regions or environments [15, 16]. Therefore, based on daily clinical practice and a large number of relevant studies and guidelines, we proposed our own pulmonary node imaging-grading and reporting system (PNI-GARS) in Chongqing, China [17]. In the early stage, we compared the diagnostic efficacy of PNI-GARS and Lung-RADS on pulmonary sub-solid nodules, which showed that PNI-GARS was superior to Lung RADS [18]. However, this study was only a single-center study with a small sample size, with only 185 pulmonary nodules, and did not include solid nodules. Therefore, we report the results of a retrospective multicenter study, as follows.

## Methods

### Patients

**Fig. 1:**
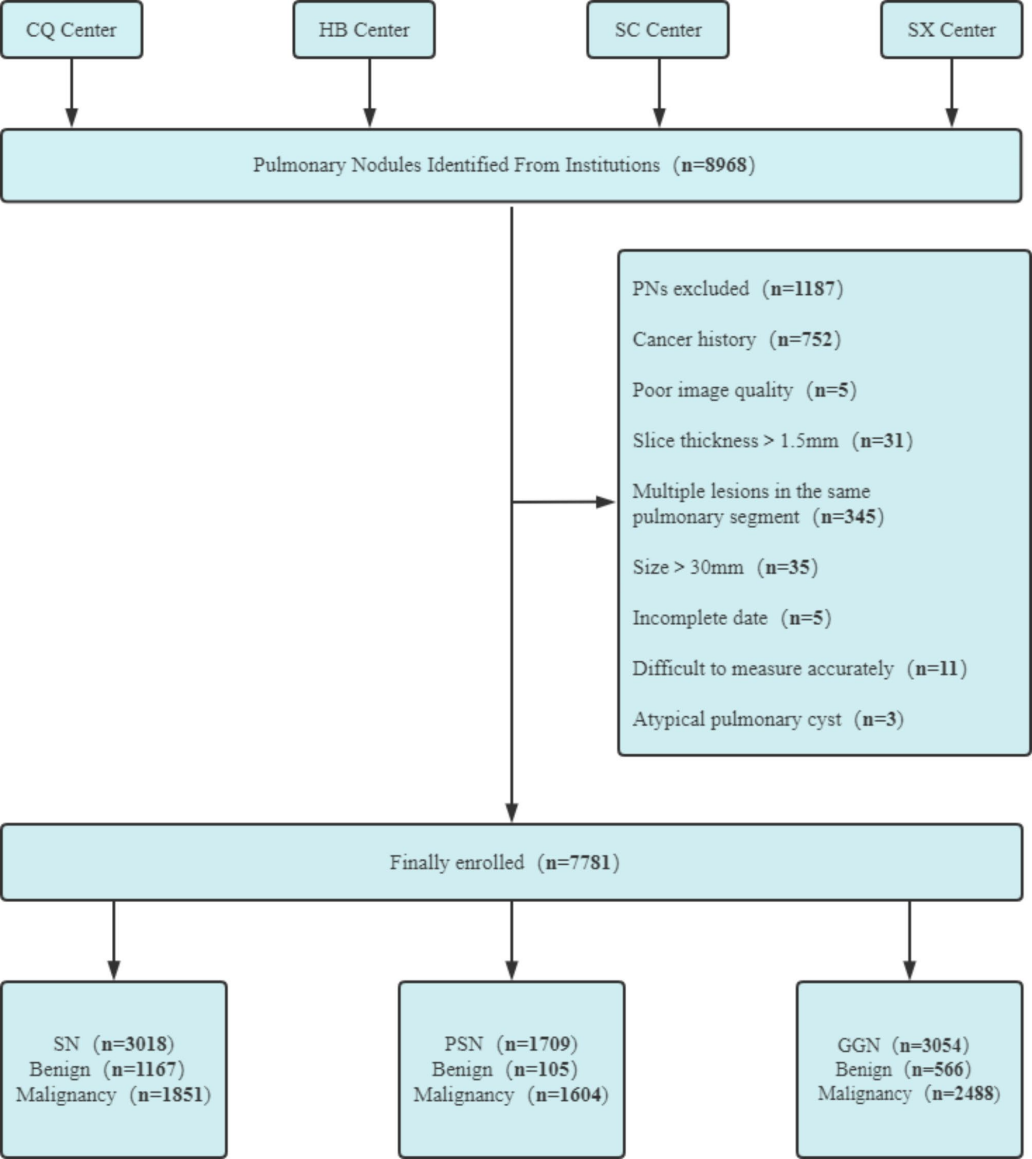
Pulmonary nodules (PNs) recruitment process at four centers. CQ Center, The First Affiliated Hospital of Chongqing Medical University; HB Center, Hubei Cancer Hospital; SC Center, Sichuan Provincial People’s Hospital; SX Center, The Second Hospital of Shanxi Medical University. SN, solid nodule; PSN, partial solid nodule; GGN, ground-glass nodule

This retrospective study was performed with approval and waiver of informed consent from the local institutional review board. All patients who underwent surgical resection of PNs were recruited from four independent centers, namely, The First Affiliated Hospital of Chongqing Medical University, Chongqing City, China, named the CQ Center during the period of January 1, 2018, to December 31, 2021; Hubei Cancer Hospital, Hubei Province, China, named the HB Center, during the period of January 1, 2021, to June 30, 2022; Sichuan Provincial People’s Hospital, Sichuan Province, China, named SC Center, during the period of September 1, 2021, to December 31, 2021; and The Second Hospital of Shanxi Medical University, Shanxi Province, China, named SX Center, during the period of January 1, 2021, to December 31, 2021, respectively. Information on the four institutions that participated in this study is shown in Fig. 1.

The inclusion criteria were as follows: the size of the PN was ≤ 30 mm; the imaging data were complete, with a slice thickness of less than 1.5 mm; patients received HRCT within 3 months before the operation; and the final pathological results were obtained after surgical resection.

The exclusion criteria were as follows: diameter of nodules > 30 mm; incomplete data; slice thickness was > 1.5 mm; images with severe noise or motion artifacts that interfered with observation; nodules were so close to the hilum that the nodule size could not be accurately measured; presence of obstructive pneumonia, atelectasis, pneumothorax, or massive pleural effusion; pathological results were not clear; patients had undergone a needle biopsy preoperatively; presence of an atypical pulmonary cyst; patients with previous malignancy; and multiple lesions in the same pulmonary segment were difficult to be accurately matched with pathology.

### CT protocol

All patients were asked to place their hands over their heads in a supine position, take a deep breath and hold their breath. The scan range was from the tip of the lung to the level of the costophrenic angle. The chest HRCT scanning parameters of different centers are shown below.

CT scanners: SOMATOM Definition Flash, Force and AS (Siemens Healthineers, Erlangen, Germany); Discovery CT750 HD, LightSpeed VCT and Revolution (GE Healthcare, Milwaukee, WI, USA). The protocol parameters: tube voltage, 80–120 kV; tube current, 10–500 mA; slice thickness, 5 or 10 mm; reconstruction slice thickness, 0.6, 1 or 1.25 mm; matrix: 512 × 512; rotation speed, 0.25, 0.5, 0.6 or 1.0 s/r; and pitch: 1, 0.984, 0.992, 1.2 or 1.9.

### Image analysis

Imaging classification of PNs: the image window width (WW) and window level (WL) were set as follows: lung window-WW 1500 Hu, WL -600 Hu, mediastinal window-WW 300 Hu, and WL 60 Hu. SN was defined as a lesion whose density was greater than that of the blood vessels and could be seen in the mediastinal window. GGN refers to the presence of low-density nodules that cannot cover the passing vessels in the lung window. PSN refers to the presence of a solid component in the nodule, with the remaining components being of ground-glass density.

The diameter of PNs was measured as follows: (I) all PNs were measured by one radiologist (CQ center: M.X.L.; HB center: Q.Y.; SC center: T.G.; SX center: K.S.) on the picture archiving and communication system (PACS) at each center; (II) the size of nodules was observed and measured on thin images, and the diameters of PNs were measured at the lung window, usually at the transverse slice, unless the longest diameter of the nodule was in the coronal or sagittal position [19]; (II) the maximum level of the nodule was selected for the measurement; (IV) both the long and short axes were measured to one decimal point, and the mean nodule diameter was reported to one decimal point [8]; (V) each nodule was measured three times to obtain its average value; (VI) millimeter was used as the measurement unit.

All PNs were classified by Lung-RADS 2022 [8] and PNI-GARS (Tables 1 and 2) by two researchers (F.P.S. & M.X.L) in a blinded manner. If the two researchers disagree, they will discuss and make a decision. In Lung-RADS, a negative screen was defined as categories 1 and 2, and a positive screen was defined as categories 3 and 4. In PNI-GARS, grades 0, I and II were defined as negative, and grades III and IV were defined as positive. Correspondingly, the pathological diagnosis of a benign nodule was negative, and that of a malignant nodule was positive.

**Table 1:** PNI-GARS

Category	Grade Score	Imaging Features	Suggestion	Risk of Malignancy
Definitely benign	0	No pulmonary nodules **OR** Pure calcified nodules, nodules with fat component, spherical atelectasis, perifissural nodules	No special treatment. Annual follow-up with LDCT for high-risk groups.	-
Benign	I	**Micronodule** with any density: ≤5 mm; **Solid nodules** > 5 mm and unchanged ≥ 2 years; **Sub solid nodules** > 5 mm and unchanged ≥ 5 years, the lesions decreased (but not increase in density) or disappeared during follow-up	LDCT 12 months, and if the nodule absorbs or shrinks, there is no need to pay attention. If the nodule increases, management is proceeded as Grade II.	-
Probably Benign	II	5 ~ 8 mm,partial edge smooth.	5 ~ 6 mm, LDCT 6months; 6 ~ 8 mm, LDCT 3 months. Nodules shrink and management is proceeded as Grade I. Nodules become larger and management is proceed as Grade IIIa. Nodules are enlarged with early malignant signs, and management is proceed as Grade IV. Nodules remain unchanged, and the follow-up time was doubled.	Very low probability
Probably Malignant	III	8 ~ 30 mm(≤ 30 mm); **Partial solid nodules** ( solid component ≤ 5 mm); **Endobronchial nodules**	Further examination or LDCT 1 month	-
	IIIa	Small nodules: 8 ~ 10 mm; GradeII nodules with malignant signs such as vacuole, vascular convergence, lobulation, etc.	LDCT 1 month	Medium probability
	IIIb	Medium nodules: 10 ~ 20 mm;Grade IIIa nodules with malignant signs such as vacuole, vascular convergence, lobulation, etc.	Tumour markers, PET/CT(solid component ≥ 8 mm), Percutaneous lung biopsy, Bronchoscope. Thoracoscopic resection is recommended if the auxiliary examination is positive or the pulmonary nodules seriously affect the patient’s life. Nodules with early malignant signs were classified as Grade IV. Endobronchial nodules are recommended for LDCT 1 month later. If there is no change, bronchoscope is recommended.	High probability
	IIIc	Large nodules: 20 ~ 30 mm;Grade IIIb nodules with malignant signs such as vacuole, vascular convergence, lobulation, etc.	The same with Grade IIIb	Very high probability
High Suspicious Malignant	IV	8 ~ 30 mm, nodules with spiculation sign or vacuole, vascular convergence, lobulation, etc. which increase the probability of malignancy; **Partial solid nodules**(solid component > 5 mm)	Surgical resection	Malignancy confirmed by imaging
Malignancy Confirmed by Pathology	V	Malignant disease confirmed by pathology	-	-

LDCT low dose computed tomography

**Table 2:** Main grading basis of PNI-GARS

Item	Details
Size	Basic standard of grading, the larger the diameter of the nodules, the higher the possibility of malignancy
Margin	Smooth: I; Partial smooth: II; Lobulation: III; Short spinous protrusion: IV
Density	Calcification: 0 (higher than pulmonary vascular, similar to ribs) Solid: I (similar to pulmonary vascular); Non solid: II(lower than pulmonary vascular, higher than pulmonary parenchyma) Partial solid: III (III ≤5 mm, IV > 5 mm);
Periphery	In the same lung lobe, there are satellite lesions: fibrosis, calcification, nodules, exudation, consolidation, etc., and the grade score is decreased one level
Endobronchial nodules	Grade III
Special signs of early malignant lesions (III: 1 signs;IV: ≥ 2 signs)	A.Vacuole sign B. Vascular convergence sign C. A solid component of GGN
Other suspicious signs of malignancy	Spiculation, GGN that doubles in 1 year or its density more than − 600 Hu, enlarged lymph nodes, size of nodule reduced but density increased during follow-up, etc.
Clinical or CT findings suggest possible inflammation	Clinical infection symptoms: A history of respiratory system infection in the past 3 months; Respiratory system symptoms have appeared at recent, and the lesions change rapidly. CT signs of inflammation: vague or unclear margin, high density in the center and ground glass density of edge, multiple lesions, satellite lesions, Tree-in-Bud sign.

GGN ground-glass nodule

### Statistical analysis

Quantitative variables are summarized by their mean or median value. The Pearson chi-square test was used to compare the difference in the diagnostic agreement rate between the Lung-RADS and PNI-GARS, and the difference in sensitivity and specificity between the two systems was compared by the McNemar χ^2^ test. Statistical significance was assumed at P < 0.05. When the sample size was < 40 or the theoretical frequency (T) was < 1, Fisher’s exact probability method was used. When the sample was more than 40 but 1 < T < 5, Yate’s correction was selected.

## Results

### General patient data

A total of 6511 patients with 7781 PNs were included in this study. There were 2815 males (58.1 ± 10.8 years, range 21–86 years) and 3696 females (55.6 ± 11.2 years, range 16–85 years). The PNs comprised 3018 SNs (benign 1167, malignant 1851), 1709 PSNs (benign 105, malignant 1604) and 3054 GGNs (benign 566, malignant 2488).

### Statistics of pathological diagnosis of PNs

**Fig. 2:**
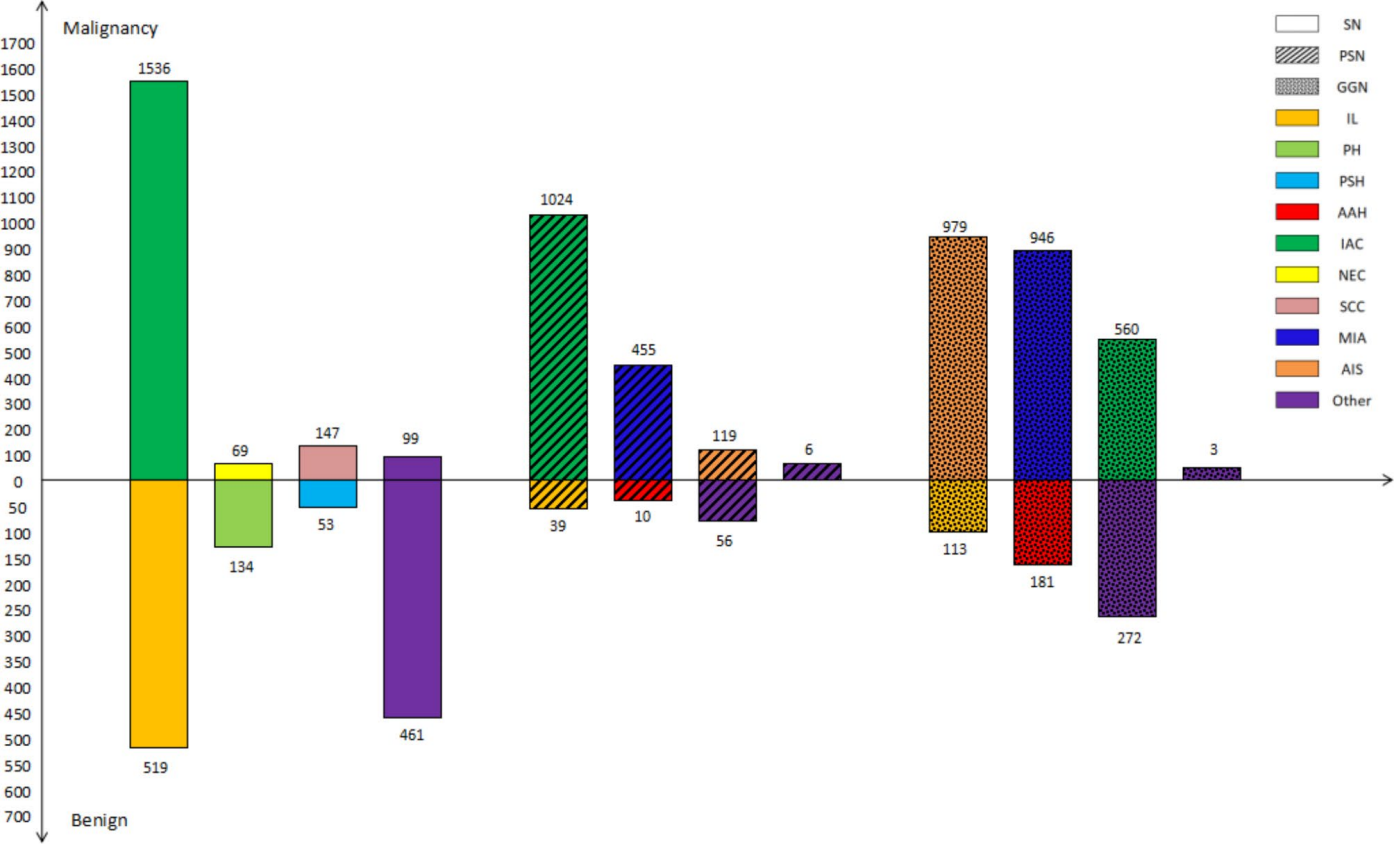
Statistics of pathological diagnosis of pulmonary nodules. SN, solid nodule; PSN, partial solid nodule; GGN, ground-glass nodule; IL, infectious lesion; PH, pulmonary hamartoma; PSH, pulmonary sclerosing hemangioma; AAH, atypical adenomatous hyperplasia; IAC, invasive adenocarcinoma; NEC, neuroendocrine carcinoma; SCC, squamous cell carcinoma; MIA, micro-invasive adenocarcinoma; AIS, Adenocarcinoma in situ

In benign PNs, infectious lesions, including tuberculosis, cryptococcus, granulomatosis, organized pneumonia and inflammatory pseudotumors, accounted for 44.43%, 37.4% and 19.96% of all benign SNs, PSNs and GGNs, respectively. For malignant nodules, the most common pathological type of SN and PSN was invasive adenocarcinoma, while adenocarcinoma in situ was the most common disease of GGN (see Fig. 2**)**.

### Comparison of Lung-RADS and PNI-GARS in the diagnostic ability of PNs

For SN and PSN, the sensitivities of PNI-GARS and Lung-RADS were similar (SN 99.8% vs. 99.4%, P < 0.001; PSN 99.9% vs. 98.4%, P = 0.015), but the specificity (SN 51.2% > 35.1%, PSN 13.3% > 5.7%, all P < 0.001) and agreement rate (SN 81.1% > 74.5%, P < 0.001; PSN 94.6% > 92.7%, P = 0.025) of PNI-GARS were superior to those of Lung-RADS.

For GGN, the sensitivity (96.5%) and agreement rate (88.6%) of PNI-GARS were better than those of Lung-RADS (0, 18.5%, P < 0.001), but the specificity of Lung-RADS was higher than that of PNI-GARS (100% > 53.7%). In general, for the entire sample, the sensitivity (98.5%) and agreement (87.0%) of PNI-GARS were greater than those of Lung-RADS (57.5% & 56.5%, all P < 0.001), whereas the specificity was slightly lower than that of Lung-RADS (49.8% < 53.4%, P < 0.003), as shown in Tables 3 and 4.

**Table 3:** Diagnostic accuracy of pulmonary nodules by lung RADS and PNI-GARS

Type	System	TP	FN	FP	TN	Se (%, 95%CI)	Sp (%,95%CI)	AR (%,95%CI)
**SN**	Lung	1839	12	757	410	99.4(99.0−99.7)	35.1(32.4–37.9)	74.5(73.0−76.1)
(n = 3018)	PNI	1848	3	569	598	99.8(99.7–100)	51.2(48.4–54.1)	81.1(79.6–82.4)
**PSN**	Lung	1578	26	99	6	98.4(97.8–99.0)	5.7(1.2–10.2)	92.7(91.5–93.9)
(n = 1709)	PNI	1602	2	91	14	99.9(99.7–100)	13.3(6.7–19.9)	94.6(93.5–95.6)
**GGN**	Lung	0	2488	0	566	0(0)	100(100)	18.5(17.2–19.9)
(n = 3054)	PNI	2401	87	262	304	96.5(95.8–97.2)	53.7(49.6–57.8)	88.6(87.4–89.7)
**Total**	Lung	3417	2526	856	982	57.5(56.2–58.8)	53.4(51.1–55.7)	56.5(55.4–57.6)
(n = 7781)	PNI	5851	92	922	916	98.5(98.1–98.8)	49.8(47.5–52.1)	87.0(86.2–87.7)

Abbreviations: *Lung* Lung-RADS, *PNI* PNI-GARS, *TP* true positive, *FN* false negative, *FP* false positive, *TN* true negative, *Se* sensitivity, *Sp* specificity, *AR* agreement rate, *CI* confidence interval. *SN* solid nodule, *PSN* partial solid nodule, *GGN* ground glass nodule

**Table 4:** Comparison of diagnostic performance between lung RADS and PNI-GARS

Type	Indicator	χ^2^	P
	Se	—	<0.001^a^
**SN**	Sp	—	<0.001^b^
	AR	22.70	<0.001^c^
	Se	5.97	0.015^d^
**PSN**	Sp	—	<0.001^a^
	AR	5.02	0.025^c^
	Se	—	—
**GGN**	Sp	—	—
	AR	3007.29	<0.001^c^
	Se	—	<0.001^b^
**Total**	Sp	—	0.003^b^
	AR	1777.76	<0.001^c^

a, Fisher’s exact probability; b, McNemar χ^2^ test; c, Pearson χ^2^ test; d, Yate’s correction; Se, sensitivity; Sp, specificity; AR, agreement rate; SN, solid nodule; PSN, partial solid nodule; GGN, ground glass nodule

## Discussion

This multicenter study revealed that the diagnostic performance of PNI-GARS was superior to that of Lung-RADS in the overall sample. However, for different types of PNs, there were some similarities and differences between them.

For solid nodules, the sensitivity, specificity and agreement rate of PNI-GARS were higher than those of Lung-RADS, and the differences were statistically significant (all P < 0.001). However, after careful analysis of the data, it was found that although the sensitivity of PNI-GARS was better than that of Lung-RADS, the difference (99.8% vs. 99.4%) was minimal. Under the premise of a large sample in our research, the difference was statistically significant but not of great practical significance, so we believed that there was no difference in sensitivity between the two systems. The specificity of Lung-RADS was inferior to that of PNI-GARS, which may be related to the following reasons. First, benign SNs were mostly infectious diseases such as tuberculosis. Previous research demonstrated that the probability of absorption or dissipation of SNs after anti-inflammatory treatment was much lower than that of PSN and pGGN, which was only 22% [20, 21]. However, these infectious SNs always tended to be large in size, corresponding to a high score according to the classification system. Due to the fear of lung cancer, surgical resection is often the first choice for patients after anti-inflammatory therapy fails. Second, in fact, due to the same fear, the interval for some patients who choose regular follow-up chest CT was often no more than three months. Nevertheless, Category 3 or 4 A pulmonary nodules needed to be stabilized for 6 months or 3 months, respectively, before they could be downgraded to Category 2 or 3. Obviously, such nodules cannot be classified as negative screens. Third, PNs with benign signs such as calcification, satellite focus, fibrosis, or peripheral exudation would be degraded by one level according to PNI-GARS regardless of the follow-up time. Therefore, some PNs at Grade IIIa would be downgraded to Grade II and become negative nodules.

Similar to the solid nodule, the specificity and agreement rate of PNI-GARS were superior to those of Lung-RADS for the evaluation of PSNs, while their sensitivity was close (99.9% vs. 98.4%, P = 0.015; there was no practical significance because of the large sample, so it was considered that they were similar). The reason for the high specificity of PNI-GARS may be related to its degradation scheme. When there were benign signs such as patchy exudation around the PSN, the PSN was graded down one level according to PNI-GARS. However, after retrospective analysis of the data, it was found that the specificity of PNI-GARS and Lung-RADS for PSN were very low, only 13.3% and 5.7%, respectively. This meant that both PNI-GARS and Lung-RADS had a high misdiagnosis rate; specifically, the probability of benign PSNs being removed by surgery was very high. However, there were only 105 benign PSNs in the present study, accounting for only 6.1% of all PSNs, so the high misdiagnosis rate of PSN would not have a significant impact on the overall population. Analysis of the corresponding data from our research showed that the proportion of definite inflammatory lesions in benign PSNs was 37.1%. Yu et al. found within their cohort that 55% of PSNs resolved during follow-up [21]. Therefore, appropriate short-term anti-inflammatory treatment for such nodules found at baseline may reduce the misdiagnosis rate, increase the specificity and reduce the risk of unnecessary surgical resection.

Comparison of the evaluation of SNs and PSNs by PNI-GARS and Lung-RADS revealed that although Lung-RADS set the nodule diameter of 6 mm as the critical point, which was lower than the 8-mm point set by PNI-GARS, the present study showed that the sensitivity of Lung-RADS was not improved but that the specificity was reduced to a certain extent. In other words, benign SNs and PSNs were more likely to be misjudged as malignancy by Lung-RADS, which would lead to a high false-positive rate, especially when its findings regarding Category 4X were not clear. This result is consistent with those of previous studies [13, 14]. For patients, this meant that the probability of unnecessary surgical resection, the medical burden and the risk of surgery were increased.

**Fig. 3:**
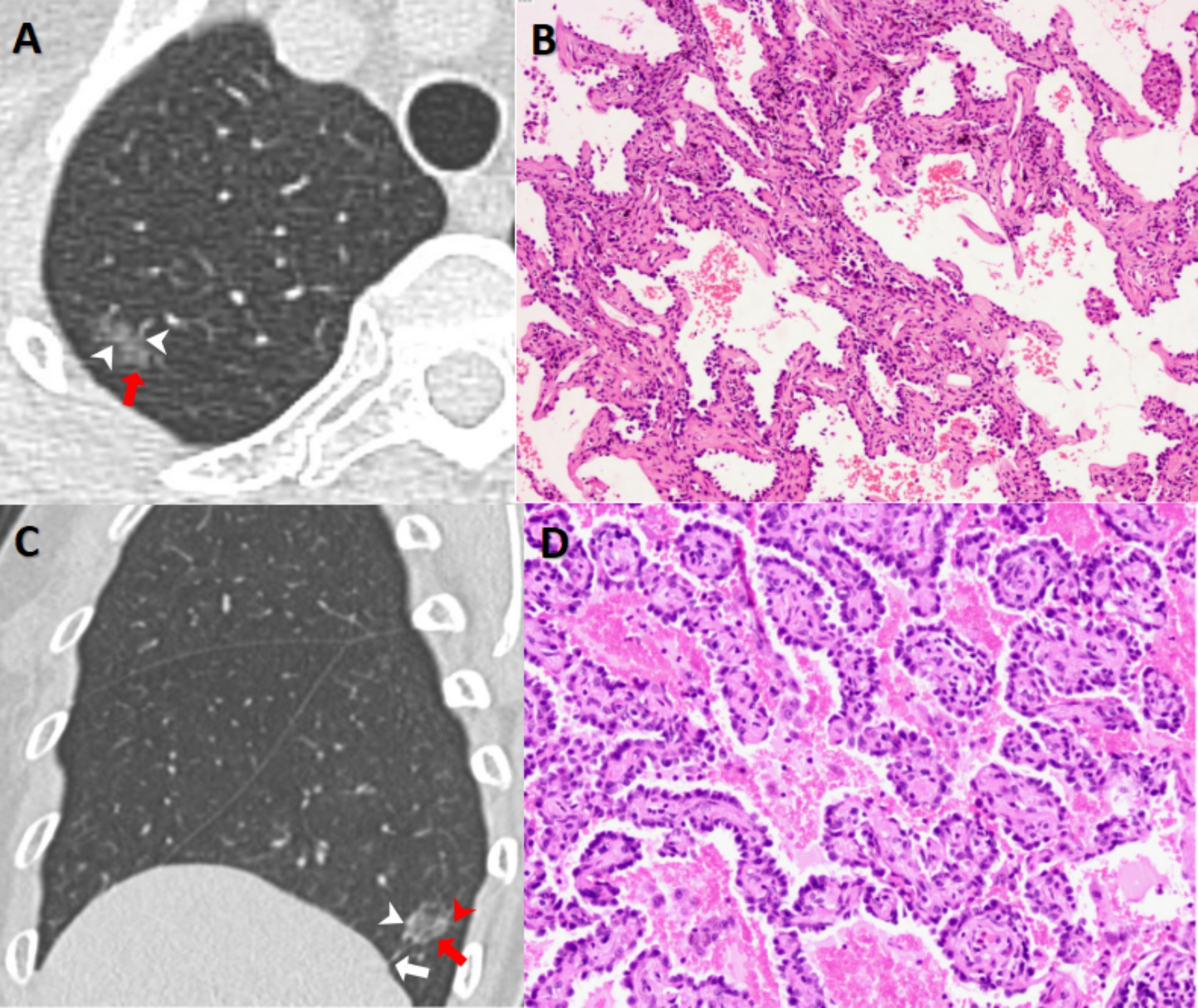
GGNs with different pathological types. (**A**&**B**, **MIA**) A GGN with an average diameter of 9.5 mm could be seen in the upper lobe of right lung (**A**, red arrows)in a 71-year-old female, and vascular shadows (**A**, white arrowheads) could be seen inside it. Its score of Lung-RADS and PNI-GARS were 2 and IIIb,respectively.The final pathological diagnosis (**B**, HE×200) was microinvasive adenocarcinoma (MIA). (**C**&**D**, **IAC**) A GGN with a mean diameter of 11.4 mm could be seen in the lower lobe of right lung (**C**, red arrows) in a 56-year-old female, and vessels (**C**, white arrowheads), spiculation (**C**, red arrowheads ) and pleural indentation signs (**C**, white arrows) could be seen. Its score of Lung-RADS and PNI-GARS were 2 and IV, respectively. The final pathological diagnosis (**D**, HE×400) was invasive adenocarcinoma (IAC)

In addition, our study also confirmed that the Lung-RADS underestimates the malignancy risk of nonsolid nodules. This was also similar to the results of previous relevant literature [11, 22–24]. Lung-RADS classified all nonsolid nodules (GGNs) with a diameter of less than 30 mm into Category 2, the descriptor of which was benign, which was inappropriate and resulted in high specificity (100%). In this research, malignant GGNs accounted for 81.5% (2488/3054) of the total GGNs, in which the proportions of adenocarcinoma in situ (AIS), microinvasive adenocarcinoma (MIA, Fig. 3A**&B**) and invasive adenocarcinoma (IAC, Fig. 3C**&D**) were 39.3% (979/2488), 38.0% (946/2488) and 22.5% (560/2488), respectively. Because there were two cells whose actual frequency was zero in the fourfold table, statistical comparison could not be performed, so our data showed that the sensitivity and agreement rate of PNI-GARS were much higher than those of Lung-RADS (sensitivity 96.5% > 0; agreement rate 88.6% > 18.5%). Consequently, PNI-GARS had high accuracy and a low rate of missed diagnosis for GGNs.

**Fig. 4:**
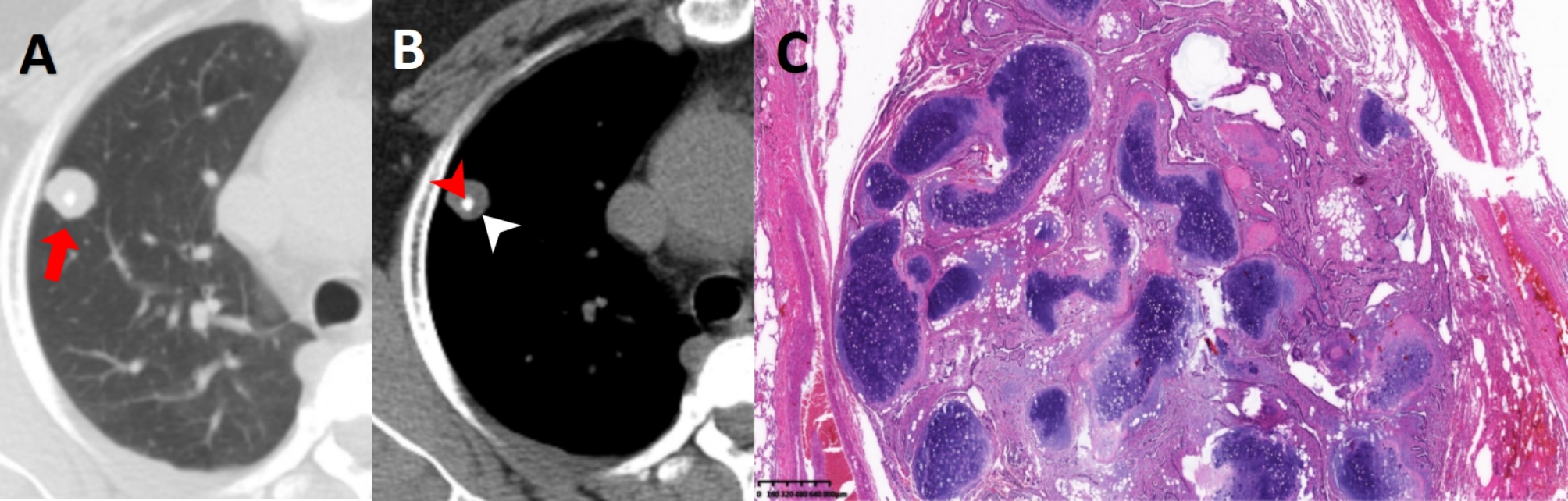
Male, 56 years old. A SN with a mean diameter of 11.0 mm could be seen in the upper lobe of right lung (**A**, red arrows), and calcification (**B**, red arrowheads)and little fat component (**B**, white arrowheads) could be seen in its center at mediastinal window. Its score of Lung-RADS and PNI-GARS were 1 and 0, respectively. The final pathological diagnosis (**C**, HE×100) was pulmonary hamartoma

Comparing the grading or category standards between Lung-RADS and PNI-GARS, we found some similarities and differences between them. The similarities include the following: first, the larger the diameter of the nodule is, the higher the risk of malignancy [23, 25]. Therefore, the diameter of the pulmonary nodule was the main criterion for the category or grading of the two systems. It was not difficult to find that the score of the category or grade of PN both increased with increasing nodule size. Second, definitely benign nodules, such as pulmonary hamartoma (Fig. 4), were classified as the lowest grade or category, which were Category 1 in Lung-RADS and Grade 0 in PNI-GARS. Finally, both systems paid attention to the malignant signs of nodules because PNs with malignant signs were more likely to be finally diagnosed as cancer [23, 26]. For both PNI-GARS and Lung-RADS, the score of category or grading of nodules with malignant signs is increased.

**Fig. 5:**
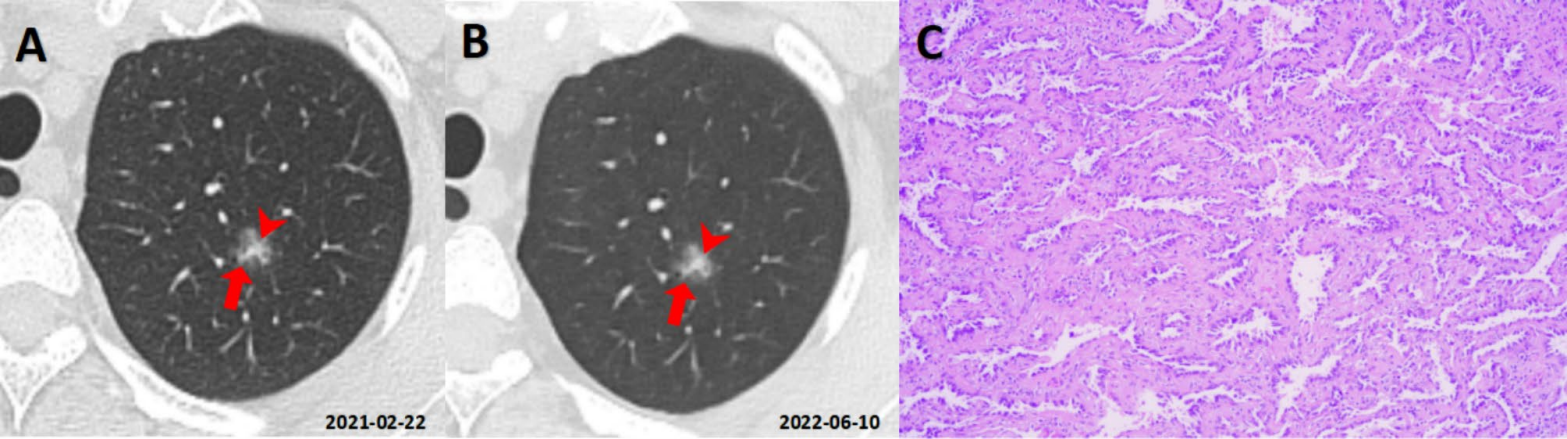
Malignant partial solid nodule stable for more than 6 months. Female, 54 years old. A partial solid nodule with a size of about 9 mm was seen in the upper lobe of the left lung (A, red arrows) at February 22nd, 2021. The solid component (A, red arrowheads) was about 4.0 mm in diameter. There was no significant change in the size of nodule (B, red arrows) and solid components (B, red arrowheads) at June 10th, 2022. Its score of Lung-RADS and PNI-GARS were 2 and IIIa, respectively. The final pathological diagnosis (C, HE×100) was invasive adenocarcinoma

**Fig. 6:**
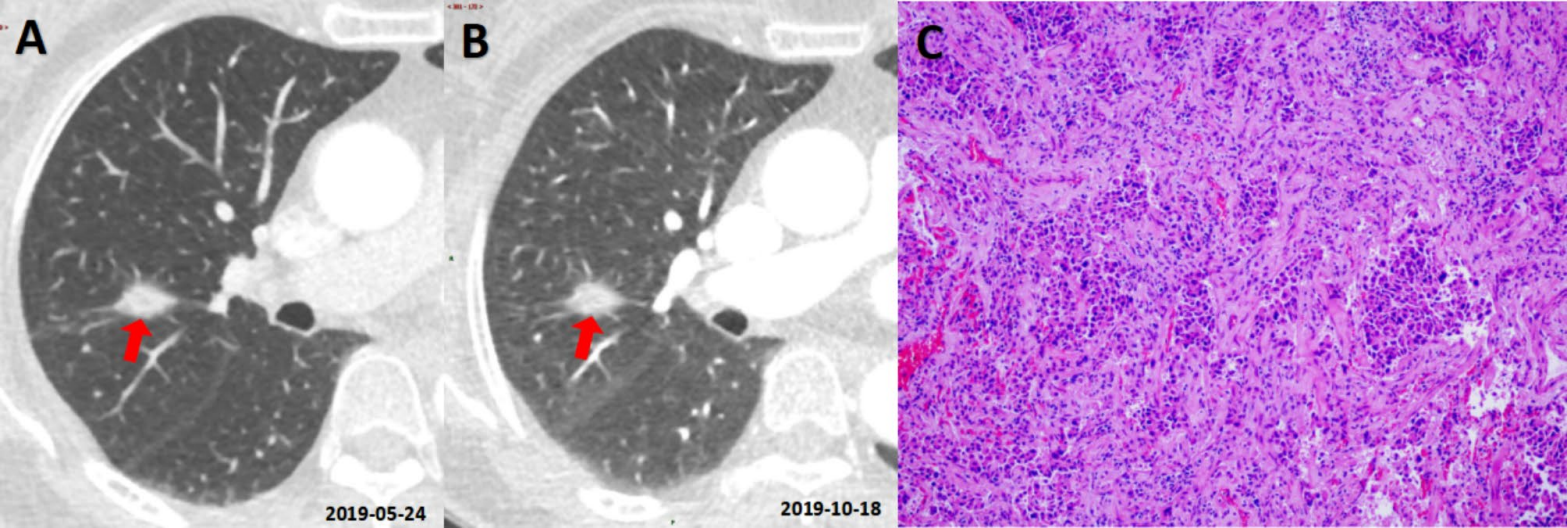
Malignant solid nodule stable for more than 3 months. Female, 60 years old. A solid nodule with a size of about 14.9 mm was seen in the upper lobe of the right lung (**A**, red arrows) at May 24nd, 2019. The mean diameter size of nodule was stable at October 18th, 2019 (**B**, red arrows). Its score of Lung-RADS and PNI-GARS were 3 and IIIc, respectively. Invasive adenocarcinoma was confirmed by pathology (**C**, HE×100)

Of course, PNI-GARS and Lung-RADS exhibited more differences. First, the upgrading criteria for nodule category were different between the two systems. The upgrading standard of PNI-GARS was relatively stable. Grade II or III nodules with one malignant sign could move up one level, and those with two or more malignant signs could move up two levels, up to Grade IV. However, the upgrading criteria of Lung-RADS were relatively vague; that is, Category 3 or 4 nodules with malignant signs were directly upgraded to Category 4X. Then, for SNs and PSNs, the diameter critical points of positive and negative nodules were different, with Lung-RADS bounded by 6 mm and PNI-GARS bounded by 8 mm. Third, for GGNs, nodules less than 30 mm in diameter were classified as negative by Lung-RADS, while 8 mm remained the boundary between positive and negative nodules in PNI-GARS. Fourth, the two systems had different criteria for nodule degradation. Lung-RADS emphasized that the follow-up time, i.e., Category 3 or 4 A nodules unchanged for 3 or 6 months, would be classified as Category 2 and 3. In addition to emphasizing the stable time of nodules, PNI-GARS also paid attention to the influence of benign signs on grading. Moreover, there were different follow-up intervals for unchanged nodules, which could be downgraded between the two systems. Lung-RADS required 3 and 6 months, while PNI-GARS required 2 years of follow-up for SN and more than 5 years for sub-solid nodules, which was consistent with the relevant literature [27, 28]. Therefore, compared with Lung-RADS, PNI-GARS was relatively conservative for downgrading by follow-up time. In our research, there were also cases of SNs and PSNs that were finally pathologically diagnosed as invasive adenocarcinoma and unchanged for more than 3 months, thus confirming the robustness of the degradation criteria in PNI-GARS (Figs. 5 and 6).

The current study also had some limitations. First, the enrolled cases were patients who underwent surgical resection, and some cases with effective anti-inflammatory therapy and long-term follow-up were not included. Therefore, there may be some selection bias. Second, because our study was a multicenter cooperative study, the four centers are located in the central, northern and southwestern regions of China, which are representative, to a certain extent, of the general population. However, the impact of regional factors on the incidence rate could not be completely excluded, and the number of centers needs to be further expanded in future research. Third, there were no evaluation criteria for atypical pulmonary cysts and GGNs with sizes greater than 30 mm in PNI-GARS, and such nodules were not included in the study; thus, some biases were inevitable. Eventually, although the study was a multicenter and large-sample study, it was retrospective. Therefore, a prospective, high-quality study with a larger population is still required to verify our results further.

## Conclusions

In conclusion, PNI-GARS was superior to Lung-RADS 2022 in the assessment of pulmonary nodules, especially nonsolid nodules (GGNs).

### Electronic supplementary material

Below is the link to the electronic supplementary material.

Supplementary Material 1

## Data Availability

The datasets used and/or analysed during the current study are available from the corresponding author on reasonable request.

## Abbreviations

PNI-GARS: Pulmonary node imaging-grading and reporting system
Lung-RADS: Lung imaging-reporting and data system
PN: Pulmonary nodule
SN: Solid nodule
PSN: Partial solid nodule
GGN: Ground-glass nodule
ACR: American College of Radiology
WW: Window width
WL: Window level
PACS: Picture archiving and communication system
T: Theoretical frequency
AIS: Adenocarcinoma in situ
MIA: Microinvasive adenocarcinoma
IAC: Invasive adenocarcinoma
