# Supplementary material for: Comparison of different classification systems for pulmonary nodules: a multicenter retrospective study in China
Source: Cancer Imaging. 2024 Jan 22;24:15. doi: 10.1186/s40644-023-00634-y (PMC10801946; doi:10.1186/s40644-023-00634-y)
Supplement: Supplementary file 1 — Supplementary Material 1 [file 40644_2023_634_MOESM1_ESM.doc]

**Comparison of different classification systems for pulmonary nodules: A multi-center retrospective study in China**

Feipeng Song^1^ Qian Yang^2^ Tong Gong^3^ Kai Sun^4^ Wenjia Zhang^4^ Mengxi Liu^1^

Fajin Lv^1^

1 Department of Radiology, The First Affiliated Hospital of Chongqing Medical University，

Chongqing, China

2 Department of Radiology, Hubei Cancer Hospital，Wuhan, China

3 Department of Radiology, Sichuan Provincial People's Hospital，Chengdu, China

4 Department of Radiology, The Second Hospital of Shanxi Medical University，Taiyuan, China

**Feipeng Song**: [15834055634@163.com](mailto:15834055634@163.com)

**Qian Yang**: 728295931@qq.com

**Tong Gong**: 786291364@qq.com

**Kai Sun**: 783611751@qq.com

**Wenjia Zhang**: 66000640@qq.com

**Mengxi Liu**: liu_mengxi@qq.com

**Corresponding author**: Fajin Lv, MD, The First Affiliated Hospital of Chongqing Medical University, Department of Radiology, No.1 YouYi Road, Chongqing, 400010, Phone: 0086-02389952228, fajinlv@sohu.com.
